# Mycobacterium Avium Complex Infection of the Spine in a Patient Without Acquired Immune Deficiency Syndrome: A Case Report and Literature Review

**DOI:** 10.1111/os.13736

**Published:** 2023-04-24

**Authors:** Hui Lv, Jian Hong Zhou, Yuan Guo, Hui Chen, Jian Zhong Xu, Zhong Rong Zhang, Ze Hua Zhang

**Affiliations:** ^1^ Department of Orthopaedic, Jiangbei Branch of Southwest Hospital 958th Hospital of the PLA Army, No. 29 Jianxin East Road, Jiangbei District Chongqing 400000 China; ^2^ Department of Orthopaedic, Southwest Hospital The First Affiliated Hospital of Army Medical University No. 30 Gaotanyan Zhengjie, Shapingba District Chongqing 400038 China

**Keywords:** Case Report, Metagenomic Next Generation Sequencing, Mycobacterium Avium Complex, Spinal Infection, Spine

## Abstract

A 52‐year‐old patient misdiagnosed with spinal tuberculosis was successfully diagnosed with Mycobacterium avium infection using metagenomic next‐generation sequencing and cured with four‐drug combination protocol chemotherapy (amikacin, rifampicin, clarithromycin, ethambutol) and spinal fixation.

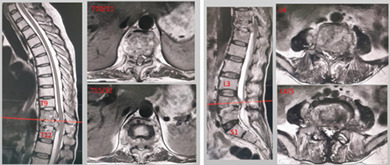

## Background

Vertebral osteomyelitis is a rare disease which remains a major challenge of treatment and diagnosis, usually caused by infections of Staphylococcus aureus and E. coli.^1^ Currently, spinal infection caused by tuberculosis is more common than ever before due to the rapid prevalence of drug‐resistant bacteria, but it is extremely rare to report the infection of Mycobacterium Avium Complex (MAC) in the spine. MAC which composed mainly of mycobacterium avium and mycobacterium intracellulare is the most common cause of nontuberculous mycobacteria pulmonary disease.^2^ Vertebral osteomyelitis‐caused MAC infection represents a major challenge in accurate and early diagnosis, because clinical signs and symptoms, as well as imaging findings resemble traditional tuberculosis infection. In this study, we report a case of hopping vertebral osteomyelitis caused by MAC in a patient without acquired immune deficiency syndrome (AIDS). In addition, MAC was successfully diagnosed by metagenomic next‐generation sequencing (mNGS).

## Case Presentation

### Therapeutic Procedure

A 52‐year‐old male was admitted to our hospital due to suffering from low back pain for more than 10 years, aggravating for half a year and progressive left lower limb radicular pain with accompanying numbness in the last 2 weeks. He had a history of high blood pressure that was well controlled. He told us his back was mildly painful about 10 years ago without any injury and no treatment was given. However, 6 months later, the patient's lower back pain worsened, and he was unable to stand because of the severe back pain. Also, the patient showed suspicious symptoms of tuberculosis, such as night sweats, slight fever, weight loss, and a string of abnormal laboratory test evaluations including raised erythrocyte sedimentation rate (ESR) and increased level of C‐reactive protein (CRP) during the last 6 months. Moreover, the pathogen samples were taken repeatedly in other hospitals, and all samples were examined by T‐SPOT, Xpert, smear and common bacterial culture and confirmed to be negative. He was eventually given diagnostic antituberculosis treatment (*Isoniazid, rifampicin, ethambutol, and pyrazinamide*), but the symptoms were only slightly relieved.

### Physical Examination and Auxiliary Examination

The physical examination at this admission revealed the spinous process and paraspinal of lumbar 4–5 vertebrae and thoracic 10–12 vertebrae were obvious tenderness and percussion pain. Decreased sensation was observed from the umbilicus down, especially on the outside of the left thigh and calf. The Laseque test was positive (about 40°) in the left lower limb and negative in the right lower limb. On motor examination, excluding the iliopsoas muscle (grade V strength), the other muscles of the left lower limb were grade IV, and the muscle strength of the right lower limb was normal. Moreover, muscle atrophy was marked in the left lower limb. Bilateral pathological signs were indefinitely positive. The pain visual analog scale (VAS) score was 8 and the Oswestry disability index (ODI) was 52 (86.7%).

**FIGURE 1:**
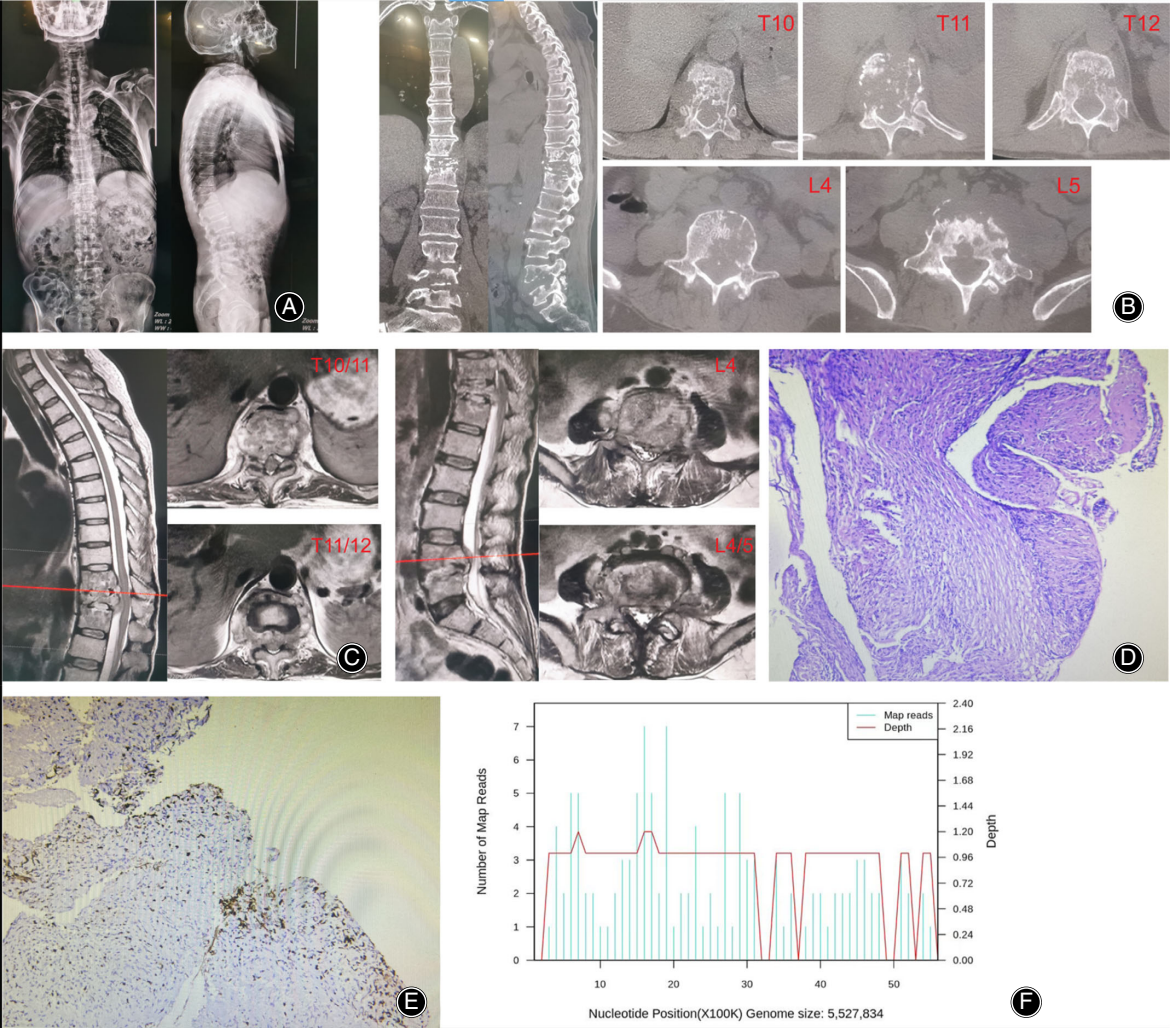
(A) The preoperative X‐ray showed wedge change in thoracic 11 vertebrae. (B) CT bone scans indicated multiple vertebral bone destruction. (C)Pretreatment MRI demonstrated multiple vertebral bone destruction and paravertebral abscess formation with significant compression of the spinal cord. (D) HE stain showed chronic inflammation with some fibrous exudationm. (E) Immunohistochemical showed acid fast stain was negative. (F) mNGS revealed the infected bacterium was Mycobacterium avium and had a genome coverage of 0.15%.

Laboratory test showed raised ESR (68 mm/h, reference range 0–20 mm/h) and CRP (22.16 mg/L, reference range 0–6 mg/L), but white blood cells and neutrophils were in the normal range. There were lower hemoglobin (106g/L, reference range 120–150 g/L) and glomerular filtration (38.6 ml/min, reference range >70 mL/min) rates. The chest CT scan displayed no apparent signs of tuberculous. X‐ray and CT scan of spine (Figure 1A,B) exhibited multiple vertebral bone destruction (thoracic 10–12 vertebrae and lumbar 4–5 vertebrae). In addition, MRI demonstrated skip bone destruction, spinal cord compression at the level of T_11‐12_ and left L_5_ nerve root compression (Figure 1C).

### Successful Diagnosis and Treatment

**FIGURE 2:**
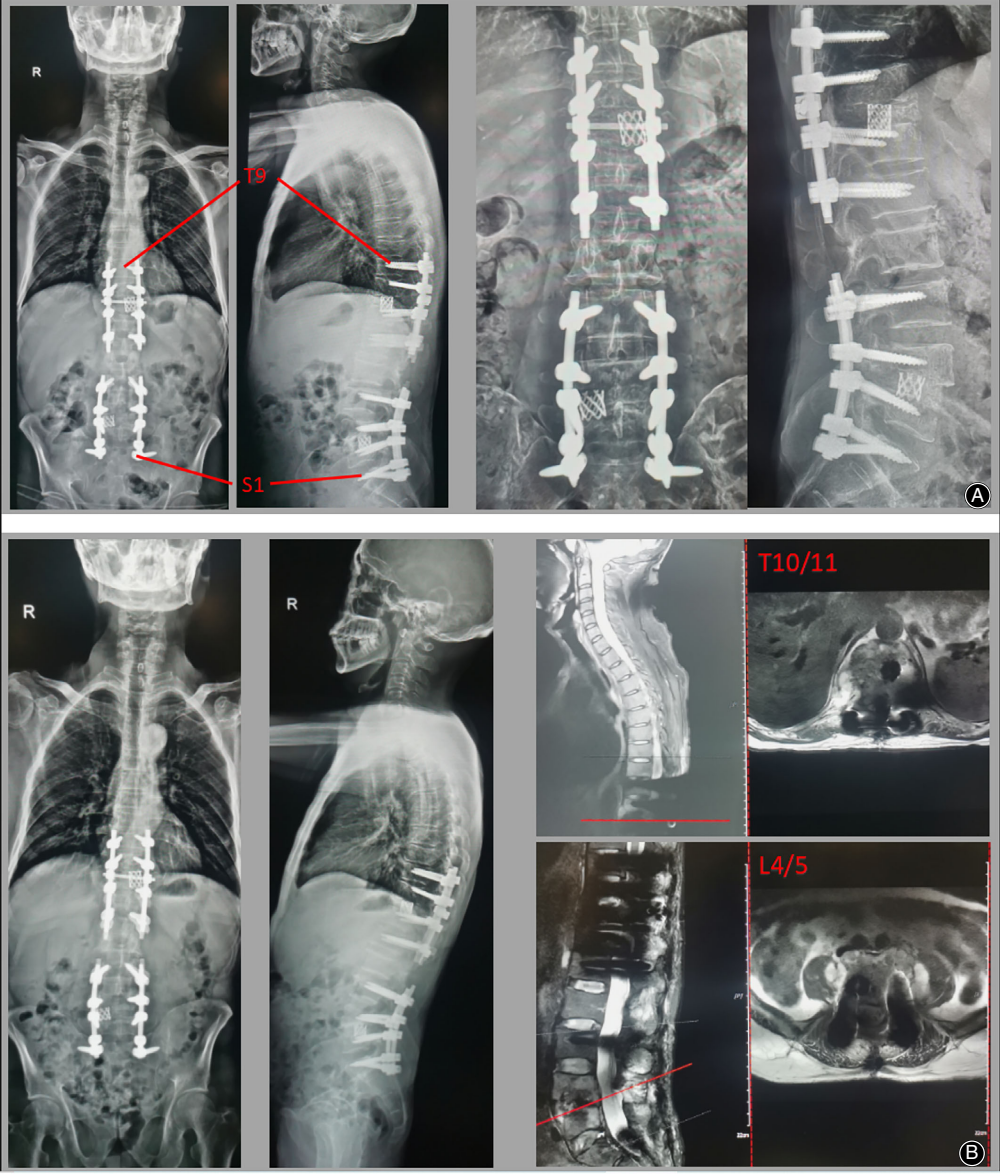
(A) The postoperative X‐ray showed the kyphosis was significantly corrected. (B) X‐ray 4 months after the operation indicated the titanium cage was fixed in place and MRI showed the spinal canal was thoroughly decompressed.

**FIGURE 3:**
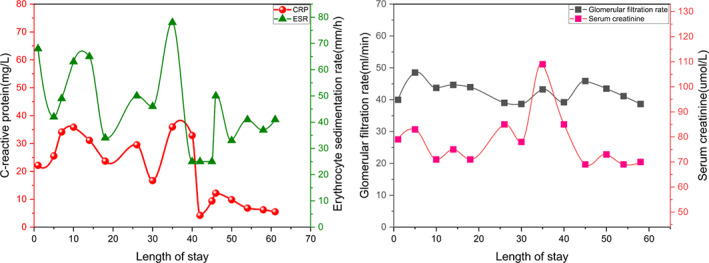
Line plot of markers of infection and changes in renal function during hospitalization.

**FIGURE 4:**
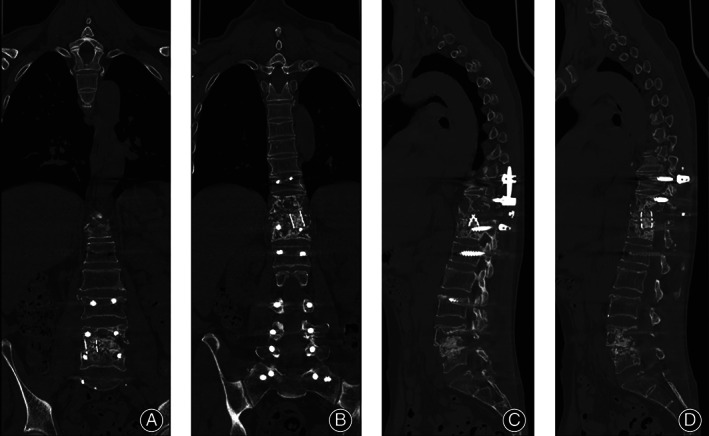
Intervertebral bony fusion was observed on CT scan at the 7‐month follow‐up.

The most important and first treatment option is to extract etiological evidence and numerous specimen retrieval operations were performed including CT‐guided percutaneous biopsy and surgical biopsy by percutaneous endoscopic lumbar discectomy. Pathological findings showed fibrous tissue with inflammatory cell infiltration (Figure 1D,E). However, the results of all the tests including tissue culture, Gram stain, and acid‐fast stain were initially disappointing. Finally, MAC was identified by mNGS (Figure 1F, Table 1). Subsequently, four‐drug regimen (amikacin 800 once every two days, rifampicin 600 mg daily, clarithromycin 500 mg twice daily, ethambutol 750 mg daily) were given for 2 weeks and all infection indicators continued to decline (CRP 16 mg/L, ESR 46 mm/h). Renal function was strictly monitored and did not fluctuate significantly under this regimen (Figure 3). After infection was controlled and general condition was improved, the patient underwent posterior thoracolumbar osteomyelitis debridement, nerve root decompression, pedicle screw fixation, and interbody fusion because of the presence of spinal mechanical instability and spinal nerve compression (Figure 2A). The next day after surgery, lower back pain caused by thoracolumbar mechanical instability was relieved, as were symptoms caused by compressed nerves (the VAS was 3), and the Laseque test of the left lower limb was negative. Infection indicators stabilized in the normal range at 4 weeks after surgery (Figure 3) and the patient was discharged from the hospital, but antibiotic treatment must continue for a minimum of 12 months. During the follow‐up of 4 months, the X‐ray films showed good fixation of the screws and rods (Figure 2B). Also, bone fusion had been detected by CT examination at the 7‐month follow‐up after surgery (Figure 4). A good therapeutic effect was obtained at a follow‐up of 10 months after discharge in this case.

**Table 1:** The results of the mNGS in the patient

Variable	Species reads	Relative abundance (%)	Genus reads	Genus reads
**Pathogenic microorganisms**				
Mycobacterium paraintracellulare	123	18.55	Mycobacterium	133
Mycobacterium intracellulare	73	11.01	Mycobacterium	133
Mycobacterium avium	1	0.15	Mycobacterium	133
**Background microorganisms**				
Burkholderia dolosa	19	2.87	Burkholderia	150
Burkholderia stabilis	17	2.56	Burkholderia	150
Burkholderia pyrrocinia	15	0.75	Burkholderia	150
Acinetobacter johnsonii	50	7.54	Acinetobacter	65
Acinetobacter parvus	3	0.45	Acinetobacter	65
Moraxella osloensis	67	10.11	Moraxella	68
Cutibacterium acnes	5	0.75	Cutibacterium	5
Pseudomonas aeruginosa	2	0.3	Pseudomonas	4

## Discussion

The most important finding of this case report is that the poor chemotherapy effect of spinal tuberculosis cannot only be limited to non‐standard chemotherapy, drug‐resistant tuberculosis, and so forth, and the possibility of tuberculosis of other strains should be carefully considered. Nontuberculous mycobacteria (NTM) is a general term for a large group of mycobacteria other than Mycobacterium tuberculosis complex and Mycobacterium leprae. Generally, the radiologic and clinical features of NTM infection are similarly to tuberculosis and this similarity may be an important reason for the high rate of misdiagnosis. Wu et al.^3^ indicated that 22.9% of patients with NTM infection had received classical antituberculosis regimens. In addition, due to the relatively unsatisfactory effects, NTM infection may be treated as drug‐resistant TB. This misdiagnosis is detrimental to the patient's condition and may lead to the generation of drug‐resistant bacteria.

### The Sensitivity of mNGS

The key to effective management of spinal infections is to identify the pathogenic bacteria in the infection microenvironment. Bacterial culture of diseased tissue has always been the gold standard for diagnosis in the clinic. Unfortunately, the positive rate of tissue culture for Mycobacterium tuberculosis, Brucella, and fungi is extremely low. Although Gray et al.^4^ asserted that repeated puncture tissue culture can improve the positive rate, there are still some disadvantages such as long culture cycle and heavy economic burden. Since the first application of mNGS in the diagnosis of clinical infection in 2008,^5^ its development has been particularly rapid in recent years. Many studies have reported that mNGS have been widely used in orthopaedic infectious diseases.^6^, ^7^, ^8^ Ma et al.^9^ showed that mNGS had a specificity of 75.0% and a sensitivity of 70.3% in the diagnosis of spinal infections. Astur et al.^10^ investigated the presence of potentially infectious bacteria in patients with disc herniation by mNGS examination. Zhang et al.^11^ reported successful finding of pathogenic bacteria by mNGS in patients with periprosthetic joint infection with negative bacterial culture and recommended routine use of mNGS in patients with bone infection. Similarly, MAC was identified in the current study by performing mNGS of paraspinal abscess and tissue. To date, MAC infection of the spine is rare, with only 17 cases reported in the English literature.^4^ The diagnosis was confirmed by biopsy and microbial culture in 16 patients, while MAC infection was determined by PCR in only one patient (Table 2). It was a fact that microbial culture was the gold standard for pathogenic detection but has been a limited method in the last decades. mNGS may play an indispensable role in spinal infections, which has the characteristics of wide pathogen coverage, rapid detection, and effective detection of dead pathogens. It should be noted that there may be a publication bias, and some MAC‐culture negative patients were missed. Additionally, seven of the patients had a long history of steroid use to control the corresponding underlying disease before MAC infection. But in this case, MAC infection with destructive changes of T_10‐12_ and L_4‐5_ in a middle‐aged man with normal immune function was first reported and this also revealed the possibility of multiple abscesses in MAC infection. Previously, several lines of evidence indicated that a string of risk factors including immune dysfunction, advanced age, osteoporosis, previous spinal surgery, and chronic obstructive pulmonary disease could increase the likelihood of MAC infection.^21^ However, owing to none of these risk factors, the initiating cause of MAC is unknown in this case, and further investigations will be needed to illustrate the underlying pathogenic factors.

**TABLE 2:** Summary of patients with vertebral osteomyelitis associated with Mycobacterium Avium complex

Author	Age/sex	Underlying cause	Involvement of vertebrae	Clinical symptoms	Diagnosis	Number of biopsies	Antibiotic regimen	Duration of drugs/ Reason for discontinuation	outcomes
Zvetina 1982^12^	35/M	SLE, SU	L1, right humeral head osteomyelitis	shoulder pain	Microbial culture	1	Isoniazid, ethambutol, streptomycin	24 mths/death	Died of aspiration pneumonia
Brodkin 1991^13^	27/F	SLE, SU, hydroxychloroquine use	L5	back pain, fever and hyporeflexia	Microbial culture	1	Rifampin, cycloserine, ethambutol, streptomycin	20 mths/death	Died of a recurrence of drug resistant abscess
Pirofsky 1993^14^	79/M	SLE, SU	Thoracolumbar with kyphosis	back pain, urinary incontinence	Acid fast stain, microbial culture	2	Clindamycin, amikacin, clofazimine, ethambutol, rifampin	6 mths/ death	Died of pneumonia
King 1994^15^	31/F	None	T7‐T8	back pain	Acid fast stain, microbial culture	1	Clarithromycin, rifampin, ciprofloxacin, cycloserine, ethambutol	24 mths/NA	Functional improvement
Mahan 1995^16^	12/F	None	Multifocal	Left leg pain	Microbial culture	1	NA	NA/NA	Pain relief but inability to walk without assistance
Igram 1997^17^	39/M	None	T6‐T7	back pain, paraplegia	Microbial culture	1	ciprofloxacin, erythromycin, ethambutol	6 mths/NA	Good recovery
									occasional back
									pain
Weiner 1998^18^	70/F	chronic bronchitis	T12	back pain, paraplegia	Microbial culture	2	Clarithromycin, rifampin, ethambutol	NA/NA	Dense neurological involvement
Chan 2001^19^	62/F	None	L5‐S1	back pain	Microbial culture	1	Clarithromycin, ethambutol	21 mths/NA	Good recovery
							clofazimine		
Niazi 2002^20^	60/F	Sarcoidosis, SU, COPD, osteoporosis, splenectomy	T7‐T9	back pain, fevers	Acid fast stain, microbial culture	1	Clarithromycin, rifampin, ethambutol	24 mths/NA	Good recovery
Mehta 2003^21^	72/F	Polymyositis, SU	T11‐L1	back pain, fevers, decreased sensation	Acid fast stain, microbial culture	1	Clarithromycin, rifampin	NA/NA	Pain relief but movement weakness
							ethambutol		
Wong 2008^22^	60/M	None	L2‐L3	back pain	Pathological histology, microbial culture	1	Amikacin, ethambutol, rifampicin	12 mths/NA	Good recovery
Takakuwa 2010^23^	76/F	None	T4‐T5	back pain, MAC pulmonary infection	Microbial culture	1	Clarithromycin, rifampicin, moxifloxacin	NA/NA	NA
Bhatia 2011^24^	70/M	ILD, SU	T5‐T7	Back pain, ataxia	Microbial culture	1	NA	NA/NA	Good recovery
Suzuki 2013^25^	67/M	DM	Thoracic, lumbar spine	back pain, fever	PCR	1	Clarithromycin, rifampicin, cycloserine, ethambutol, streptomycin sulfate	24/NA	Good recovery
Shimizu 2013^26^	38/F	SLE, SU	T8‐T9	back pain	Microbial culture	1	Clarithromycin, rifampin	12/NA	NA
							ethambutol		
Shimizu 2013^26^	50/M	None	L2‐L5	back pain	Microbial culture	1	Clarithromycin, rifampin	12/disappearance of clinical symptoms	Good recovery
							ethambutol		
Gray 2018^4^	70/F	SLE, SU, splenectomy, AHA	L2‐L4	progressive back	Microbial culture	3	Azithromycin, ethambutol	12/NA	Good recovery
				pain					

Abbreviations: AHA, autoimmune hemolytic anemia; COPD, chronic obstructive pulmonary disease; DM, Diabetes mellitus; ILD, interstitial lung disease; NA, not available; SU, steroid use.

### Choice of Chemotherapy Regimen

On account of its rarity, there are still no guidelines in the management of spinal MAC infection. Although we have found the pathogenic bacteria, the drug susceptibility test is still not available. Therefore, we chose an empiric therapy scheme based on antibiotic regimens in MAC‐pulmonary disease.^27^ The most important antibiotics in the management of MAC infection was Macrolides, such as azithromycin, clarithromycin.^28^ However, in the treatment of tuberculosis, isoniazid and rifampicin are the first‐line bactericide. Macrophages are an important immune system to defend against the invasion of mycobacteria, but they are also the host cells of mycobacteria. Intracellular infection is a challenge that conventional single antibiotics cannot solve, so long‐term treatment and multi‐drug combination are the critical factors to prevent the recurrence of pathogens. The higher concentration of macrolide antibiotics in macrophages compared to plasma,^29^ as well as the immunomodulatory properties,^30^ play a crucial role in the management of MAC infection. Instead, the role of rifampicin, a classic first‐line anti‐tuberculosis drug, in the treatment of MAC infection is controversial.^29^, ^31^ The exact reason is not clear, but the possible interpretation may be that rifampicin significantly reduces the plasma concentration of clarithromycin when co‐administered with rifampicin and clarithromycin.^32^ Given the complexity of this patient's condition, rifampicin was administered to reduce macrolide resistance after careful communication with the patient.^29^ Although our treatment course is still less than 12 months, the process of bone fusion is difficult to proceed in the inflammatory environment of infection and the patient has returned to the community to perform simple works without the discomfort previously described. We therefore considered the treatment in this case to be successful at present. However, oral antibiotics are still needed to avoid secondary infection caused by reactivation of intracellular MAC. In fact, the successful treatment of this patient confirms that the guidelines for pulmonary MAC infections are instructive for spinal infections and the four‐drug combination protocol can be referenced.

In conclusion, this case reported MAC infection of the spine in a patient who was diagnosed successfully by mNGS, we strongly believe that mNGS may play an important role in the clinical diagnosis of pathogens that cannot be found by repeated puncture cultures and has a promising future to accurate and early diagnosis of spinal infection. In addition, in cases with poor response to diagnostic anti‐TB therapy, the possibility of MAC infection should be considered. Moreover, the four‐drug combination protocol (amikacin, rifampicin, clarithromycin, ethambutol) can achieve satisfactory results in the management of spinal MAC infection.

## Author Contributions

Hui Lv, Jian Hong Zhou participated in the conception and drafted the manuscript. Ze Hua Zhang, Zhong Rong Zhang contributed to study design, and revision of the manuscript. Jian Hong Zhou participated in the conception, study design, and draft of the manuscript. Hui Chen was responsible for the acquisition of data and the follow‐up examinations in the hospital. The authors have read and approved the final manuscript.

## Funding Information

This work was sponsored by Army Medical University Outstanding Talent Pool Key Support Object Personalized Training Project (No. 2019rcpy05) and Chongqing Talent Program (No. CQYC202105037).

## Conflict of Interest Statement

The authors declare that they have no competing interests.

## Ethics Statement

The present study was approved by the institutional review board of Jiangbei Branch of Southwest Hospital, 958th Hospital of the PLA Army.

## Data Availability

The datasets generated and/or analysed during the current study are not publicly available due to limitations of ethical approval involving the patient data and anonymity but are available from the first author (Hui Lv) on reasonable request.
